# 735. Malaria Chemoprophylaxis Adherence Among U.S. Active Duty Service Members during Deployment to Endemic Regions

**DOI:** 10.1093/ofid/ofab466.932

**Published:** 2021-12-04

**Authors:** Ryan P Collier, David A Lindholm, Tahaniyat Lalani, Kalyani Telu, Huai-Ching Kuo, Jamie Fraser, Anuradha Ganesan, Anjali Kunz, Charla Geist, Heather Yun, Heather Yun, Drake Tilley

**Affiliations:** 1 Brooke Army Medical Center, Ft Sam Houston, Texas; 2 Uniformed Services University of the Health Sciences, Ft Sam Houston, Texas; 3 Infectious Disease Clinical Research Program, Bethesda, MD; 4 The Henry M. Jackson Foundation, Bethesda, MD; 5 Naval Medical Center Portsmouth, VA, Portsmouth, Virginia; 6 Henry M. Jackson Foundation for the Advancement of Military Medicine, Bethesda, MD; 7 IDCRP, Bethesda, Maryland; 8 Infectious Disease Clinical Research Program/Uniformed Services University, Bethesda, Maryland; 9 Infectious Disease Clinical Research Program and the Henry M. Jackson Foundation for the Advancement of Military Medicine and Walter Reed National Military Medical Center, Bethesda, MD; 10 Madigan Army Medical Center, Tacoma, WA; 11 Landstuhl Regional Medical Center, Landstuhl, Rheinland-Pfalz, Germany; 12 Brooke Army Medical Center, Department of Medicine, Uniformed Services University of the Health Sciences, San Antonio, TX; 13 Naval Medical Center San Diego, San Diego, California

## Abstract

**Background:**

Military members frequently deploy to malaria-endemic regions. Most cases of travel-related malaria occur due to prophylaxis non-adherence, impacting mission readiness. Factors assessing adherence are described in outbreak settings; we prospectively assess adherence in military travelers.

**Methods:**

TravMil is a prospective, observational cohort study of US military beneficiaries traveling outside the US (2010-2019). Our analysis includes only active-duty service members traveling with a military purpose to malaria-endemic regions, who were prescribed malaria prophylaxis, and who completed a pre- and post-deployment survey; they could also enroll after return from deployment. All travelers received pre-travel counseling. Survey responses were assessed using descriptive statistics and multivariate regression to determine risk factors for adherence.

**Results:**

1504 travelers were included (85% male; median age 28 years; 73% white). Median duration of travel was 77 days (12% traveled ≤ 14 days). Africa was the most common destination (33%). Primary prophylaxis included doxycycline (54%) and atovaquone/proguanil (43%). 969 (64%) were fully adherent to their regimen. The frequency of prophylaxis did not match expected values, as 3.6% of subjects reported taking prophylaxis weekly, and 2.9% did not know how often they took it. 103 (6.9%) did not take any of the prescribed regimen. On multivariate analysis, deployers were more likely to adhere if they traveled for ≤ 14 days or to Africa or practiced other mosquito-avoidance behaviors. Study enrollment post-deployment was associated with decreased odds of adherence, as was use of a tent. The use of daily versus weekly prophylaxis was not associated with a difference in adherence, though we had limited subjects prescribed weekly regimens.

Figure 1. Reasons for not taking any of the prescribed chemoprophylaxis (n = 103)

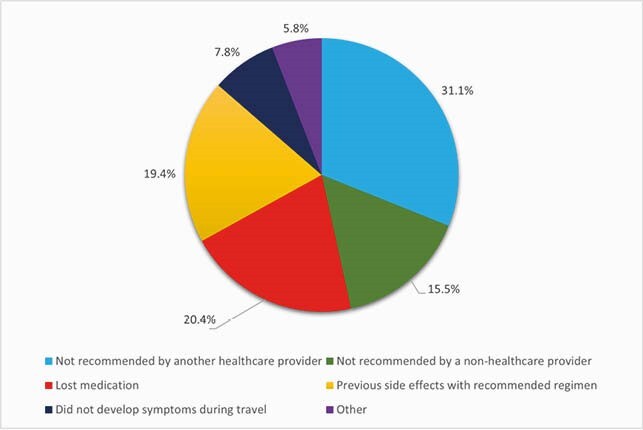

Table 1. Odds of full adherence to malaria chemoprophylaxis on multivariate logistic analysis

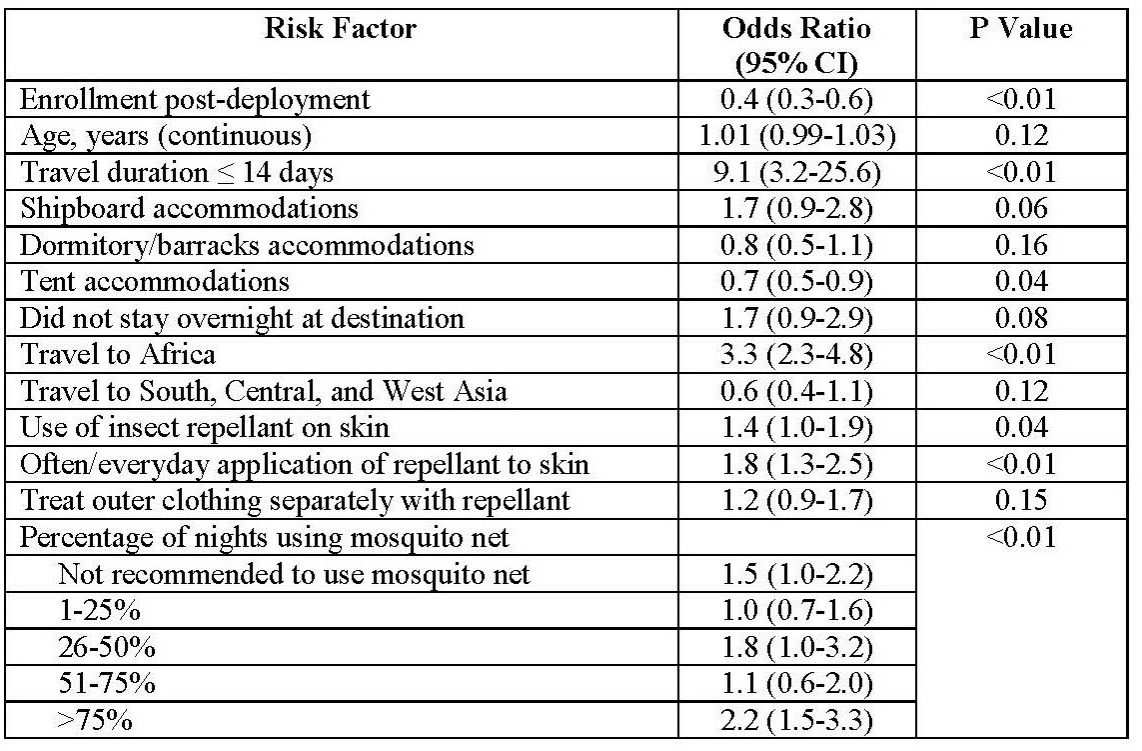

**Conclusion:**

Short-duration travel, travel to highly endemic regions, and mosquito-avoidance behaviors were associated with increased adherence to prophylaxis. The lower rate of adherence in post-deployment enrollees may be a surrogate for inadequate counseling or recall bias. Our study highlights potential holes in counseling regarding malaria prophylaxis and the importance of ongoing provider and patient education on malaria.

**Disclosures:**

**Heather Yun, MD**, American Board of Internal Medicine (Individual(s) Involved: Self): Board Member

